# Physiological Foundations for Religious Experiences in Devotional Worship Practices with Music Using Heart Rate and Respiration Rate Analyses

**DOI:** 10.3390/ejihpe12020011

**Published:** 2022-01-27

**Authors:** Yoshija Walter, Andreas Altorfer

**Affiliations:** 1 Laboratory for Cognitive Neurosciences, University of Fribourg, 1700 Fribourg, Switzerland; 2 Translational Research Center, University Hospital of Psychiatry and Psychotherapy UPD, University of Bern, 3000 Bern, Switzerland; andreas.altorfer@upd.unibe.ch; 3 Institute for Management and Digitalization, Kalaidos University of Applied Sciences Zurich, 8050 Zurich, Switzerland

**Keywords:** religion, religious experience, worship, music, heart rate, respiration rate, breath frequency, physiology, psychobiology of religion

## Abstract

The present study investigates the psychophysiological activation patterns of religious experiences in worship practices using Heart Rate (HR) and Respiratory Rate (RR) analyses. For this, 60 evangelical individuals participated in an experiment where they worshipped to six selected conditions and continuously indicated how strongly they sensed what they believed to be the presence of God. These ratings were correlated with the biometric data to indicate whether the experience has an activating effect on the believer’s vegetative system (activation hypothesis) or a soothing effect thereupon (pacification hypothesis). Statistical analyses showed that the psychological disposition during the religious worship experience speeds up the physiological responses, which was indicated by increases in HR and RR. Hence, the activation hypothesis was accepted, and the pacification hypothesis was rejected.

## 1. Introduction

In the 1970s and 1980s, a perspective called *biogenic structuralism* arose to bridge the gaps between the neurosciences, anthropology, and phenomenology. It assumed that many features typically discussed and described by psychologists, sociologists, and the humanities are in principle predisposed by genetic functions and eventually the organization of the nervous system. For the proponents of the theory, it was clear that all things cognitive had to emerge from the brain and that the physiology of the brain itself was dictated by its structural basis. The idea had the seeming advantage that it could dismiss a dualistic world view, a perspective that may be argued to have made the scientific study of mental phenomena much more complicated instead [1]. It was unifying in the sense that many different topics from an array of disciplines could be subsumed in one line of research—the study of the brain and its structural variants. Since it was primarily a biological idea, the theory was strongly influenced by evolutionary thought, especially since our hominid encephalization was generally assumed to be key for the emergence of distinct human qualities, such as communication, learning, social interactions, and mentation. Although structuralism had its advantages, it was not unilaterally well received since it seemed rather simplistic to assume that external and interaction factors would not play equally significant roles in the development of human thoughts, actions, and experiences [2,3,4,5].

Eugene D’Aquili was one of the founders of biogenic structuralism. Together with Andrew Newberg, the neuroscientist who coined the term *Neurotheology* [6,7], he later published *The Mystical Mind*, trying to dissect and explain the biological correlates of religious experience [8]. Although their work had countless critics, it demonstrated the interest that has long been present to the study of biological foundations of religious experience [5,9,10,11,12,13].

Thus far, researchers have put a decisive emphasis on the neurological study of such special states of mind. It must be noted, though, that this kind of research was invariably scarce:

At present, only a small number of studies exist on the relation between neural activity and religious behaviour, and the majority of these have focused on how religiously inspired techniques of meditation modulate practitioners’ states of consciousness.

[14] (p. 311)

To date, this situation has not changed immensely, and the main methods focus on near-infrared spectroscopy NIRS, electroencephalography EEG, and structural or functional magnetic resonance imaging s/f-MRI [15,16]. There is, however, not much known about general physiological attributes and correlates of religious states of mind, even though such studies are equally necessary to understand a given human experience. The present study thus attempts to contribute to closing this gap by measuring some core physiological variables, namely the heart rate (HR) and respiratory rate (RR).

It has proven to be advantageous to study religious experiences in the lab under the influence of music [17], since it may help with a believer’s focus on God, which is an integral part of the induction of an experience where a person feels to be in contact with the divine. Worship music can help in the facilitation of this process, and it can be adequately manipulated in the lab [18]. Music can help facilitate certain desired states of consciousness, and there is already a respectable history in the study of how music can influence biological variables in these states, especially in the field of neuroscientific studies [19,20,21,22,23,24,25]. 

Sound has the power to raise emotions [26], and it can be linked to religious experience [17]. There are also non-neural physiological variables that have been tested in musical cases, such as heart rate (HR) or respiratory rate (RR), in order to see if certain states activate or deactivate a person’s physiological system [27,28,29]. One of these protocols for analyzing HR and RR has been closely observed for the present study [29].

Whereas experience is an important dimension in a believer’s construct system of religiosity [30], it is extremely difficult to do justice to the adequate operationalization of a complex construct such as *religious experience* [31]. Since there are many intercultural nuances that must be respected if the construct is taken at large, it is advised to observe the subjective elements at play in the experiences. Hence, a religious experience can be viewed as an experience *deemed religious* by the believers themselves, and when working experimentally, it makes sense to limit the theoretical framework to a specific sample with a more or less unified dogmatic system in order to avoid a plethora of confounding factors [32,33,34]. Therefore, in the present study, we deal with the specific case of a religious experience where people believe to be sensing the divine while assuming to be having an encounter with God. For this, a clearly delineated evangelical sample was recruited.

Kölsch and Jäncke [27] have previously discussed that HR and RR are higher under the influence of exciting music as opposed to tranquilizing songs. However, music can also be used as a proxy to induce a religious experience in worship [17,18]. When participants are asked to rate their experience of the divine under such conditions, it is not yet clear if this is associated with a more activating effect or with a more relaxing one. As such, two mutually exclusive hypotheses were formulated.

The first hypothesis is the *activation hypothesis*. It states that, upon the perception of experiencing the presence of the divine in worship devotionals, an activation of the system occurs, which becomes evident through increased HR and RR. The alternative hypothesis is the *pacification hypothesis*, which effectively claims that the reverse occurs. Upon a religious experience during worship, the participants calm down, which is shown in decreased HR and RR.

## 2. Materials and Methods

### 2.1. Participants and Questionnaire

We performed empirical experiments in the lab with 60 individuals from evangelical churches. It was ensured that all participants had a comparable theological background in terms of their religious experiences by asking them about their views and experiences beforehand (in a previous qualitative study [18], it was found that a believer’s denomination is a strong predictor of theological convictions). The mean age was 27 years (SD 4.22; min 19; max 40), and the gender ratio was roughly equal (45% male; 55% female). Most of them were right-handed (87%), and the majority reported being very musical (70% said that they play an instrument more than once a week). The highest education was evenly distributed (22% had a master’s degree, 23% had a bachelor’s, 22% went to high school, and 33% have undergone an apprenticeship). All subjects had to be able to induce an experience in worship with music where they assumed that they experienced the presence of God. A hearing test was applied to exclude hearing deficiencies. All respondents filled out a questionnaire beforehand. It included questions about their prayer lives, temperament, and religious experiences (the questions were as follows: How often do you praise God *with* music? How often *without* music? How often do you sense God’s presence in worship? How often do you feel restless? How strongly can you endure doing nothing? How important is worship for your prayer life? Additionally, what do you feel when you experience God in worship? This last question allowed multiple answers that dealt with sensing something emotional or physical, receiving a divine message, sensing the divine presence, feeling close to God, and becoming happy or sad). The questionnaire also included the *ten-item personality index* (TIPI) for calculating the personality dimension using the five-factor model [35] and some questions about the respondents’ demographics. Informed consent was provided before each experiment started. The study was approved by the local Swiss ethics committee of the Canton of Bern (under the project ID number 2021-00022). Due to a system malfunctioning, one participant had to be excluded from the data. 

### 2.2. Data Acquisition

#### 2.2.1. Biometric Assessment

There were two biometric measures that were recorded during the tasks:
**Respiration rate (RR)**A cloth band was spun around the chest of the participants. The band was interwoven with a device that measured the strain applied to it. Based on the contraction and expansion of the chest, the RR was registered and calculated as cyclic measurements to analyze the number of breaths per minute.**Heart rate (HR)**HR measurements were taken from the electrocardiogram (ECG) recordings to obtain an adequate assessment of beats per minute. In addition, finger pulse measures were taken from the left index finger. Three ECG electrodes were placed in a standard fashion on the left chest. HR was calculated as cyclic variables to obtain the beats per minute at the heart (R-wave) and at the finger (maximum of pulse wave).

The biometric measurements were extremely sensitive to bodily movements, and therefore, they were prone to generating artifacts. As such, outliers were calculated and removed via a standardized algorithm: the acceptable range of values was defined as the interquartile range IQR (which is the third quartile Q_3_ minus the first quartile Q_1_) and multiplied by a factor of 1.5 to generate the upper and lower limits. All values that exceeded this upper limit (Q_3_ + IQR × 1.5) and the ones underscoring the lower limit (Q_1_ − IQR × 1.5) were counted as outliers and thus replaced by the next nearby values within the accepted range.

#### 2.2.2. Experiential Assessment

The religious experience was measured with a bar slider on the right-hand side. To avoid any further input stimuli, during worship, participants were asked to close their eyes and to continuously slide the bar up and down, depending on how strongly they believed they felt the presence of God at any given moment. After each condition, participants were asked to evaluate how strongly they were able to focus on God during this condition.

### 2.3. Experimental Design 

The experiments are constructed as a within-subject design where all participants went through the same conditions. Since there was not a random assignment of participants to groups defined by an independent variable, some readers may prefer the term “quasi-experimental” for our approach. 

Each participant took part in an experiment in the lab that lasted for about an hour. The experiment started and ended with a resting state condition. In between, there were six experimental conditions that appeared in a randomized fashion (hence, for each individual, the order of the conditions was different). All of the conditions (apart from the *empty* condition *B* and the resting state) were songs played from a speaker.

The conditions were separated by a “letter task”, which was a distraction task specifically designed to enforce the person’s diligent concentration so that the spiritual state of mind in the experimental condition was disrupted. With this, we were able to warrant that there was no spillover effect of experience from one condition to the next. As the conditions were randomized and mentally clearly set apart, the individual conditions can be treated as independent observations.

Based on Huber and Huber’s religiosity model [30] and evidence on religious experience in worship [36,37,38] (pp. 5 & 64), we were convinced that the phenomenon is best studied with a mix of worship conditions where the participants can “let go” and sink into a given state of mind, instead of producing a spiritual result by force of effort. To achieve this, a subset of the songs for achieving the desired state of mind was selected by the participants themselves and a subset was given to them as preselected (given) songs by the researchers. The given songs were selected as part of a previous study in order to pick the ideal songs in this experiment for the sample at hand [18]. Hence, the subjectively selected songs were different from participant to participant and the given ones were the same for all of them.

In order to contrast the potential variance of such a religious experience in worship with adequate comparisons, the conditions were selected according to three categories where each category consisted of two songs: religious songs, secular songs, and control conditions. For both the religious and the secular category, there was each one song selected by the participants themselves and one song pre-selected by the researchers. Table 1 summarizes the experimental conditions that were used in the current investigation. 

**Figure 1:**
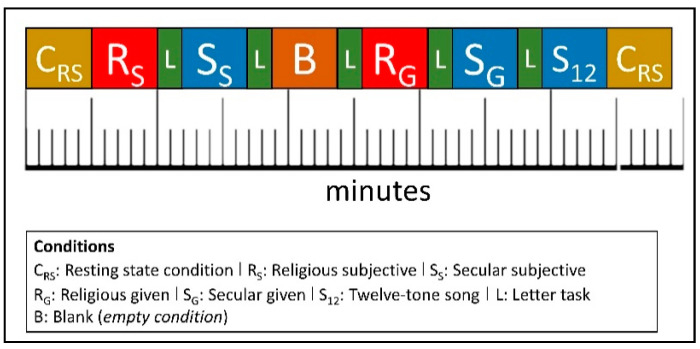
Experimental alternating block design (inspired from: [43]).

We employed an *alternating block design* where the seven conditions were compared against each other [39] (the two resting state sessions C_RS_ counted as one by using the averaged values of both conditions). A similar design with blocks ranging from 3 to 20 min was successfully employed in several meditation studies [40,41,42]. Figure 1 illustrates how the different conditions, which are described in Table 1, were placed in an exemplary experimental procedure (although, as stated above, the order of the conditions was randomized across participants).

The participants were advised to try to worship and to induce the experience during each of the conditions, except during the letter tasks and the resting states. Each condition took about 4.5 min [44]. The songs that were longer were cut at natural breaks so that they amounted to 4.5 min, and if they were shorter, they were sound engineered with Audacity 2.4.2. to naturally end at 4.5 min (by, for example, inserting a chorus or a verse twice).

With this procedure, we followed the suggestion of previous studies working with expert populations of spiritual phenomena in the construction of within-subject conditions [45,46]. 

The pre-selected songs consisted of the tune “Reckless Love” by Cory Asbury (2017, Bethel Music) for the R_G_ condition, the famous radio-version “Lose You To Love Me” by Selena Gomez (2019, Interscope Records) for the S_G_ condition, and the song “Pierrot Lunaire” by Arnold Schönberg (1874–1951, Op.21: No. 1–4, Mondestrunken, Columbine, Der Dandy, eine blasse Wäscherin) for the S_12_ condition. 

### 2.4. Statistical Analyses

#### 2.4.1. Analytical Procedures

After the data generation, the statistical analyses were performed using IBM SPSS Statistics 27. There was a step-by-step procedure followed in order to understand the data at hand, to test the hypotheses, and to shed further light on the dynamics at hand. First, the data were inspected with an exploratory approach and inspected visually in order to obtain a first understanding on how the present cohort relates the variables of interest at hand. This lays the ground for the steps that come thereafter. Second, the main question was answered by testing the two hypotheses of whether the religious experience was accompanied by an activation or a deactivation of the physiological system of the participants. Third, after answering the main question, we wanted to better understand the associations discovered and therefore analyzed if there are statistical differences of the RR and HR in the different conditions. Fourth, since there are frequent discussions about gen der differences in religiosity [47], we wanted to know whether the associations found in steps two and three were also covariately influenced by the individual’s gender. Fifth, up until this point, the sample was treated as a monolithic group. However, since there was an interesting variance in the data at play, we set up a grouping analysis where an independent component analysis showed if the sample could be split up in meaningful groups. Table 2 summarizes the different steps in the analyses made.

#### 2.4.2. Analysis 1: Exploration of the Physiological Data

The data were inspected visually to spot if the physiological values corresponded to norm values of what could be expected and whether the RR and HR followed a normal distribution pattern. A parametric Pearson correlation was used to see if the average HR and RR were aligned. The evaluation (the focus on God), which was assessed after each condition anew, was correlated with nonparametric Spearman’s Rho correlations for the maximal peak values of the religious experience to determine if the two were related as expected. The experimental conditions were sorted according to the mean of their reported experiential values and transformed into a variable with an interval scale. The conditions were then correlated with the peak experience using Spearman’s Rho. At the same time, the same correlations with the religious experience were also performed on the average HR and RR. 

#### 2.4.3. Analysis 2: Testing the Hypotheses

The hypotheses were tested by using the peak religious experience values per experimental condition and by comparing them to the physiological variables of interest, namely RR and HR. This was conducted using a regular parametric Pearson correlation and followed up with a nonparametric Spearman’s Rho correlation between the evaluation and the RR as well as the HR. A positive correlation between the religious experience and the physiology would indicate that stronger sensations of the divine presence were accompanied by higher respiratory and heart frequencies, which would mean that there is merit to the activation hypothesis. A negative correlation would imply that there was a deactivating association since stronger experiences would correlate with decreased RR and HR, which would favor the pacification hypothesis. If there would not be a significant result, then no statement about the possible relationships could be made. 

#### 2.4.4. Analysis 3: Discerning the Conditions

**Figure 2:**
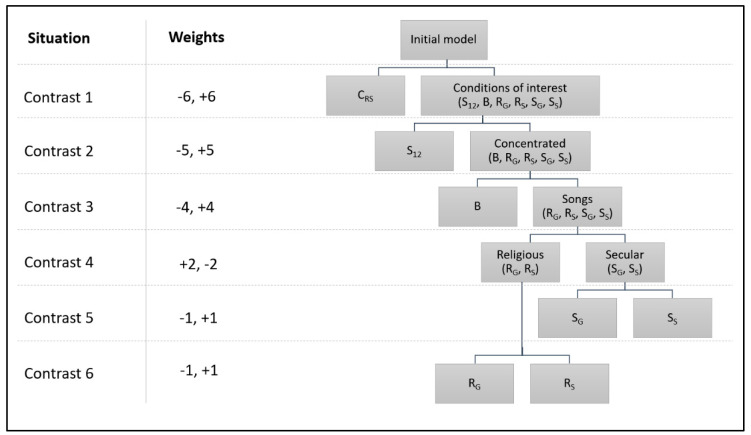
Stepwise procedure of the contrast analysis following up the ANOVA. For the sake of completeness, both physiological variables were included.

After understanding whether there was an association between the religious experience and the physiology, it was interesting to see if there was a statistical difference in the physiology across the experimental conditions. They were selected to increase the experiential variance, but these dynamics need to be discerned given the present sample. As such, an analysis of variance was attempted, although first, there needed to be a test to verify if the homogeneity of variance assumption was met. This dictates how the ANOVA must be dealt with. Hence, first, a Levene’s test was performed, and second, an ANOVA was calculated whereby the physiological variables were inserted as the fixed factors and the conditions were the dependent targets. The analysis of variance shows that at least two conditions significantly differ from the mean of all conditions, which acts as a first indication that a further follow-up with a contrast analysis would be warranted. Upon the significant ANOVA for at least one fixed factor, a planned contrast analysis was employed to see which conditions differed from each other and how they did so when considering the physiological variables. The analysis used six contrasts that were coded as follows:

The contrasts analysis was applied to discern in which conditions exactly the physiology differed. The relevant effect sizes were calculated and extracted as *Cohen’s d* values. Figure 2 displays the contrasts and weights used during the analysis, which effectively consisted of a set of standardized two-tailed T-tests. The weights of the conditions in Figure 2 are drawn from Table 3.

#### 2.4.5. Analysis 4: Covariance Analysis

**Figure 3:**
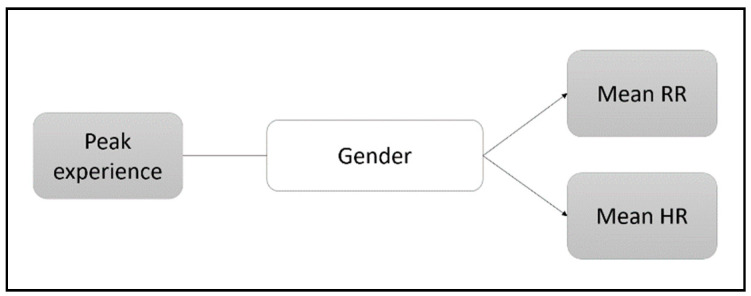
Conceptual diagram of the covariance analysis (MANCOVA) applied to see if gender was also involved in the relationship between the peak experience and the physiological variables.

As stated above, there is evidence claiming that the centrality of religiosity is not necessarily equally distributed between the genders [47], and we were interested to find out whether the influence the religious experience has on the believer’s physiology is covariately determined by a person’s gender. Therefore, a multivariate analysis of covariance was applied. As with the above mentioned ANOVA, there was a Levene’s test applied to see if the homogeneity of variance assumption of the MANCOVA was met. The covariance analysis using the MANCOVA in analysis four can be conceptualized as depicted in Figure 3.

#### 2.4.6. Analysis 5: Analyzing the Groups

Since there was a respectable variance in the data, it was interesting to figure out if the sample in our study could be split into meaningful groups. Hence, a factor analysis was performed using a principal component analysis (PCA) as an extraction method. Naturally, an oblique rotation method would be preferred since some of the variables are intercorrelated. However, since the rotation does not alter the data itself and any rotation in the present case did not yield a visible improvement for understanding the resulting factor solutions, a model with no rotation was eventually preferred. The decision on the number of factors was based upon a scree plot and conceptual considerations. A Kaiser–Meyer–Olkin (KMO) test was applied to analyze the explained variance of the factorial model and a Barlett’s sphericity test was used to determine if the variables are an adequate fit for the resulting model. For the display of the solutions, no factor loadings were suppressed so that the readers are confronted with all the results from the factor model (see Table 4). 

## 3. Results

### 3.1. Analysis 1: Exploration of the Physiological Data

The experimental conditions were separated from each other by the letter tasks as a distraction and by randomization. This allowed them to be treated as independent observations and the degrees of freedom were thus deduced from the number of participants multiplied by the observations minus the missing values. 

The RR and the HR roughly follow a normal distribution curve, and both physiological variables lie within what is generally accepted as “norm values”, which for RR, lies between 10–20 breaths per minute and, for HR, lies within the range of 60–90 beats per minute [48].

**Figure 4:**
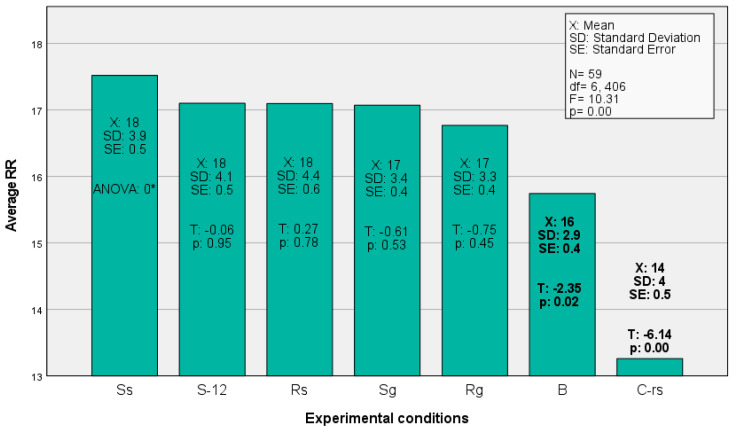
Average RR displayed by experimental condition. (* The ANOVA in S_S_ was zero due to its calculated redundancy for the constant term in the model).

**Figure 5:**
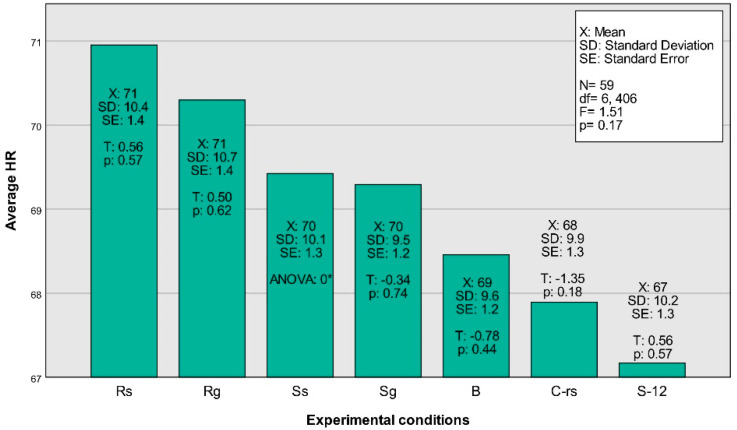
Average HR displayed by experimental condition. (* The ANOVA in S_S_ was zero due to its calculated redundancy for the constant term in the model).

The average RR and the average HR were significantly correlated, *r*(470) = 0.223, *p* < 0.001. Figure 4 and Figure 5 show that the believer’s system speeds up during the religious and secular worship conditions. It is unclear from these illustrations alone, however, if there are interesting statistical differences between the individual conditions.

As was to be expected from previous research [18], the evaluation of how strongly the participants were able to focus on God was strongly correlated with the maximal ratings of the religious experience per condition, *r*(531) = 0.791, *p* < 0.001. In other words, the more the people reported to be able to focus on God, the deeper their peak religious experience during the worship exercise became. The maximal experience itself was positively associated with the experimental condition, *r*(531) = 0.580, *p* < 0.001, indicating that depending on the experimental condition, the felt experience with God became more intense. Physiologically, there was a strong effect from the condition on the average RR, *r*(531) = 0.381, *p* < 0.001, and a weaker one on the average HR, *r*(531) = 0.119, *p* = 0.006.

### 3.2. Analysis 2: Testing the Hypotheses

Both the activation as well as the pacification hypothesis are geared toward one central question: Does the biological system of a person become activated or deactivated under the influence of an intense religious worship experience? The best proxy for the intensity of the peak religious experience was the maximal values provided by the participants’ right-hand bar slider. 

The statistical analyses showed that there were significant positive correlations for all variables of interest in this regard. The maximal rating was positively associated with both the average RR, *r*(531) = 0.334, *p* < 0.001, as well as with the average HR in each condition, *r*(531) = 0.170, *p* < 0.001. Since the peak experiences were significantly correlated with the evaluation (the focus on God), the evaluation was also positively associated with the average RR throughout all seven conditions, *r*(531) = 0.333, *p* < 0.001, and with the average HR, *r*(531) = 0.131, *p* = 0.002. 

To summarize, the religious peak experience was positively correlated with the mean RR and HR, which means that there was a physiological activation at play. The activation hypothesis may therefore be accepted, and the pacification hypothesis may be rejected.

The following three analyses were devised to better understand these dynamics.

### 3.3. Analysis 3: Discerning the Conditions

An analysis of variance (ANOVA) using the mean cyclic variables in the physiology (HR and RR) grouped by the different conditions was performed. It showed whether there were any significant differences in HR and RR across the conditional situations. The nonsignificant Levene’s test (for the HR: *F*[8, 522] = 0.147, *p* = 0.997; for the RR: *F*[8, 522] = 1.755, *p* = 0.084) allowed us to consider the model under the homogeneity of variance assumption. The ANOVA for the RR proved to be significant, *F*(8) = 15.838, *p*< 0.001, indicating that at least two of the conditions portrayed a stochastic difference between the sample means. This was not true with the HR, for which the model was nonsignificant, implying that the HR did not significantly differ if compared across the conditions.

Turning to the planned contrast analysis (see Figure 2), for the mean RR, contrast one, *T*(522) = 7.049, *p* < 0.001, and contrast three, *T*(522) = 2.549, *p* = 0.011, proved to be significant. Both effect sizes were positive with *d* = 5.9 for contrast one and *d* = 1.5 for contrast three. Although the overall model (ANOVA) of the HR was nonsignificant, a closer inspection through the contrast analyses revealed that the mean HR showed a tentative difference in contrast two, *T*(522) = 2.126 *p* = 0.034, which was also positive in its effect, *d* = 1.5. Since there were six contrasts performed, the critical alpha of 0.05 would be reduced to 0.008 if calculated by the Bonferroni error correction method for multiple testing.

In other words, for the mean RR, there was a clear difference between the resting state and the conditions of interest as well as an interesting trend between the empty condition B (worshipping without music), and the religious as well as secular songs. The RR was lowest during the resting state, and for the religious (R_S_, R_G_) as well as the secular songs (S_S_, S_G_), the rate was significantly higher than during the blank condition B. This indicates that the control conditions and different contrasts have been adequately selected. For the HR, there appeared to be a trend between the 12-tone song that was selected as a distraction and the rest of the conditions where the participants apparently had an easier time to fall into the worship experience.

### 3.4. Analysis 4: Covariance Analysis

Being able to accept the activation hypothesis means that there was a positive effect from the peak experience (maximal ratings of the religious experience) to the physiology. We were then interested in finding out whether the participant’s gender influenced this relationship as a covariate. As such, a multivariate analysis of covariance (MANCOVA) was calculated with the peak experience as the fixed factor, gender as a covariate, and the mean physiological values as dependent variables.

The homogeneity of variance assumption was not violated, as indicated by the nonsignificant Levene’s test (for the RR: *F*(288, 242) = 0.731, *p* = 0.995; for the HR: *F*(288, 242) = 0.796, *p* = 0.969). The multivariate test based on Wilks’ Lambda was significant for the participant’s gender, *F*(2, 240) = 4.981, *p* = 0.008, suggesting that the model was influenced by this covariate. The tests for between-subject effects was not significant for RR, but it was significant for HR, *F*(1) = 5.887, *p* = 0.016, which means that the heart activity was affected by a person’s gender in the context of a religious experience.

To summarize, the religious experience had an activating effect on a believer’s physiology. The effect on one’s HR was also covariately influenced by a person’s gender, whereas the effect on the respiratory system was not governed by sex determination.

### 3.5. Analysis 5: Analyzing the Groups

It is possible to statistically split the present sample into valuable groups. For this, a dimension reduction using a factor analysis with the inclusion of interesting experiential, biometric, personal, and psychological variables was performed. A principal component analysis was used as the extraction method, whereas no orthogonal or oblique rotation was undertaken.

The explained variance of the factor model was acceptable but not extraordinary, *KMO* = 0.549; however, Barlett’s test for sphericity demonstrated that the input variables were an adequate fit for the dimensions at hand, *X^2^*(120) = 2395.120, *p* < 0.001. Most importantly, the extraction communalities showed a high factor loading on the variables dealing with the religious experience. This was most visibly the case for the maximal rating (peak experience), loaded with 0.751; the experimental conditions, loaded with 0.708; and the evaluation (focus on God), correlated with 0.879 on the factorial model (cf. Table 2). For the extraction method, a scree plot and conceptual reflections led to the decision of extracting the three factors from the data.

The factor analysis, as portrayed in Table 4, suggested extracting three groups of people that can be labelled “high experiencers”, “mid experiencers”, and “low experiencers”. The high experiencers (HEs) had very high religious experiences in worship, and consequently, they were able to focus on God well. They portrayed a strong physiological activation reaction. They were, however, not as experienced in the practice as one would expect given the strength of the experience. They were rather restless, but nonetheless, believed that they can sit still. These people were emotional, open for new experiences, and characterized by some stubbornness.

The mid experiencers (MEs) lay somewhere in between since they had a mediocre focus on and experience of God during worship. They were calmer than the high experiencers, worshipped more regularly, and experienced God in worship more constantly but less intensely. They were rather introverted, conscientious, and emotionally stable but liked to keep things as they were (hence, not terribly open to new experiences).

The low experiencers (LEs) had a difficult time focusing on God and were the least likely to have an intense religious experience during the worship practice. Despite their difficulty to let go and to sink into a deep experience, they appeared to be more traditional since their worship frequency lay between those of the mid and high experiencers. Their experience of the divine presence was much more frequent and constant than with the HEs but a lot less intense. However, their experience was not as constant and intense as with the MEs. They were highly extroverted and open to the world but not as emotional and conscientious—hence, they appeared to be adventurous pragmatics.

In summary, the following picture can be drawn for the three groups of worshippers:
*HEs are similar to artists*Impulsive and intense, fast, and deep experience but not constant and not seeking it regularly*MEs are similar to athletes*Constantly working on it, steady and stable, and have deep experiences but not as extreme and volatile.*LEs are similar to business economists*Very pragmatic, open to new things, do not pursue it for very long and do not dive in very deep emotionally.

**Figure 6:**
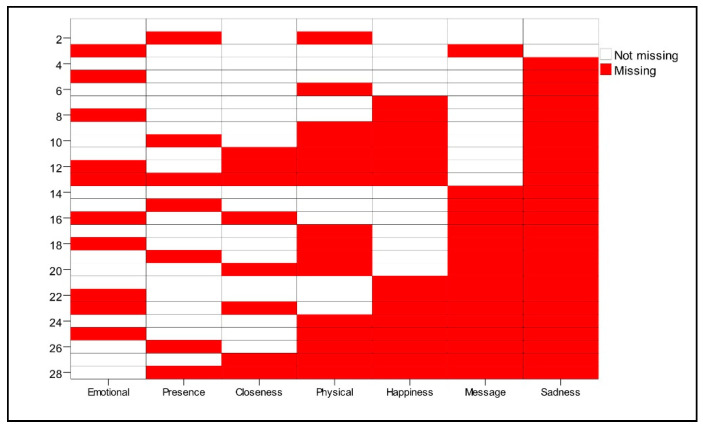
Depiction of missing values with the colored boxes (participants were asked to tick the type of religious experiences they usually experience during worship).

**Figure 7:**
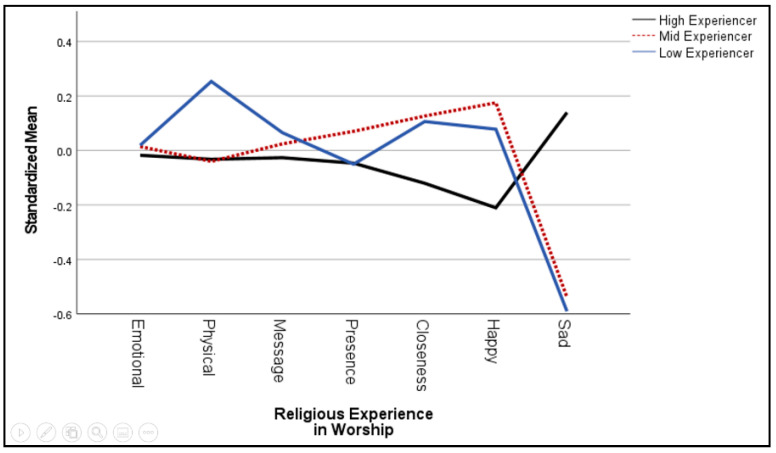
Indication of which types of so-believed divine experiences were present for the three groups of worshippers.

As visible in Figure 6 and Figure 7, HEs were the only ones with an inclination toward associating their religious experiences with a certain degree of melancholy. They were not likely to differentiate their experiences on a spectrum of different types of experiences. There was no strong difference made between the physical, epistemic, and emotional experiences. They appeared to be interested in having a strong and deep encounter with the divine, but to them, it was not important what the experience resembled. MEs had predominantly positive associations when thinking about their divine experiences. Most of the time, they experienced their religious encounters as a sensation of closeness with God, which can also be described as sensing God’s presence. LEs were the ones most likely to refer to physical experiences of the divine, which were also remembered with positive emotions. They appeared to have the strictest definition of what counted as a religious experience in worship because they primarily expected to have such physical sensations. Perhaps this was exactly why they were “low experiencers”, since they had difficulty becoming emotionally involved in the experience and only accepted more spectacular, physical encounters as real experiences with the divine.

## 4. Discussion

People who can focus on God during their practice appear to have a deeper religious experience when engaging in worship. It comes along with an activation of their physiology, as evidenced by increases in RR and HR. This effect holds true for practice with music, but it is also present when no music is used. Depending on how well believers can focus on God, their experience becomes more intense, which also manifests on the level of their vegetative nervous system.

Previous studies have shown that exciting music is linked to higher RR and HR [27]. As such, one might have assumed that the experience during practice is a derivative function of music used during the experiment, suggesting that the experience and its biological correlates are simply mirroring an activation of the tune. This idea can be rejected for two reasons: (i) first, there is a large enough variability in the subjective songs brought along by the participants that, although the music helps facilitate the experience, it is not the upbeat character of a song that provokes a deep experience and an activation of the system but the mental stimulation it provides in terms of its religious content; (ii) second, the experience and physiological activation are visible during the blank (empty) condition, when no music is played and the participants worship God in silence. Had it predominantly been a musical effect, then the blank condition would not yield a deep experience and might show a physiological deactivation. However, the opposite was observed in the present study.

The experimental worship conditions have a strong influence on how intense the religious experience is deemed to be, and the correlations show that the HR and RR are both affected in an activating fashion. For this effect to occur, it is not necessary that one is a highly proficient and frequent worshipper. As a matter of fact, in our sample, the reverse was the case: the HEs had the most intense experiences and a strong physiological reaction, but at the same time, they were the ones who practiced worship devotionals the least frequently. This was a surprising discovery since one might have assumed that more practice results in deeper experiences. These findings were further strengthened by the fact that the frequency of worship variables, the importance of worship for one’s faith, and the general proclivity to instantiate such phenomenal states were negatively correlated with the depth of the experience and hence the physiological response.

The strongest differences in the biological measurements were seen in the application of the control conditions. The songs (including the blank condition B) showed an increase when compared with the resting state, and the same was true when they were compared with the 12-tone song S_12_, which was deliberately selected for its harmonic dissonances to make achieving the worship experience more difficult. Likewise, the religious songs proved to provide a stronger activation than when people worshipped God without music, although the activation was still clearly more tangible than with the 12-tone song and the resting state. Surprisingly, the comparison between the religious and the secular songs, or the subjectively selected ones and the songs that were preselected by the researchers, did not yield any interesting physiological differences.

The effect itself was partly modulated by the person’s gender, but as seen in the factor analysis, the three groups of worshippers primarily differed not in their sex but in their personality structures. Whereas the HEs appeared to be highly empathetic and receptive for short and strong spiritual stimuli, the MEs appeared to be more traditional, calm, and willing to stay on track regularly long-term. The LEs, on the other hand, seemed to be rather pragmatic, open to new things, and willing to give them a try but were not motivated enough to search for the experience as much as the MEs would. However, unlike the HEs, the LEs were not as prone to experiencing an intense religious experience where they might be less in control over what happens internally.

These findings are in consonance with the previously published *Feedback Loop Model for Worship Experiences* [18] and they enrich the model significantly. They add to it the knowledge that these experiences come along with physiological activation patterns and that the worshippers—based on their experiences, personalities, and physiological responses—can be split into three conceptually valuable groups: the high, mid, and low experiencers, which all appear to have a somewhat different approach when it comes to their religious experiences in worship.

## 5. Conclusions

The present study intended to find out whether a believer’s peripheral physiology, as measured by HR and RR, is activated (activation hypothesis) or deactivated (pacification hypothesis) under the influence of a religious worship experience. The current analyses showed that the activation hypothesis can be accepted and that the pacification hypothesis must be rejected. This means that an intense religious experience in worship comes along with a significant activation in a person’s physiological system, which is immanent in an increased HR and RR. The five analyses reported in this publication attempt to shed light on the dynamics of this effect.

The first analysis was an exploration of the physiological data, where it was seen that HR and RR were significantly correlated. The values of how strongly the participants were able to focus on God were strongly associated with the intensity of the experience as well as with the physiological variables. The second analysis tested the hypotheses, where it was found that the physiological system of a believer speeds up under the influence of the experience, which was seen in the positive association of the religious experience with HR and RR. The third analysis looked at differences across the experimental conditions, where it was found that, for the RR, the conditions of interest differed starkly from the resting state. The RR was lowest during the resting state, and it was significantly higher with the religious and secular songs when compared with the empty condition B. This indicated that the control conditions were adequately selected. Likewise, for the HR, the 12-tone song showed higher values than the religious as well as the secular songs. The fourth analysis demonstrated that the influence of the religious experience on the peripheral physiology was covariately influenced by a person’s gender (only for HR). The fifth analysis performed a factor analysis and showed that the sample can be split up into three statistically valuable groups, namely the high experiencers, the mid experiencers, and the low experiencers.

The limitations that emerge in this research mostly relate to conceptual difficulties that are twofold: first, an experimental study must constrain possible confounding factors by keeping the design monolithic, reproducible, and unambiguous. It also implies that the recruited participants need to have a common dogmatic and socio-religious background so that a shared understanding of the idea of “worship” and the required “experience of God’s presence” can be guaranteed. If this standard is not met, then it cannot be guaranteed that a comparable state of mind is measured and analyzed for each of the participants in the different conditions. In this case, any statistical analysis would be rendered meaningless. As such, as interesting as it would be, it was not possible to make this an interdenominational, interreligious, or intercultural study. It is limited in the sense that it deals with *one* type of religious experience emerging from one clearly delineated sample. Opening the experiment to more diverse samples would automatically implicate stronger confounding factors, which in turn would diminish both the validity as well as the reliability of the study. Second, a major difficulty in the field of religious phenomenology is to have an adequate operationalization of a construct as complex as *religious experience*. To make progress and not become stuck on mere definitions, we adhere to the suggestion to take the intersubjective conceptions of the given sample seriously and to work things out from there. This does not imply that the current results are not generalizable, but it means that such a discussion must be handled with due care.

Future studies can remedy this by using a similar design and by applying it to different denominations and religions. The design should be adapted to do justice to the respective sample, and eventually the different analyses could be compared and qualitatively analyzed to generate a broader understanding of such phenomena. There is, however, also still much to be learned from a study comparable with ours: further analyses could be performed by adding different measures to the pool, such as using galvanic skin response, HR variability, and brain state studies.

## Figures and Tables

**Table 1:** Experimental conditions applied in the present study.

Acronym	Name	Content
**R_S_**	Religious subjective	This was a religious worship song provided by the participants. They sent it in beforehand so that it could be integrated into the experimental procedure. It had to be a song that they knew worked well for inducing a religious experience where they believed to be sensing the presence of God. This song was different for all participants.
**R_G_**	Religious given	This was a religious worship song preselected by the researchers based on previous interviews to select which song would work best in the denominations of the present sample for the induction of the experience. This song was the same for all participants.
**S_S_**	Secular subjective	This was a secular song that the individuals really liked and associated with comparable sentiments as with the R_S_ song. Hence, the major difference between these two from an emic perspective was the religious content in the lyrics and the associations that are connected therewith. This song was different for all participants.
**S_G_**	Secular given	This was a secular song that was selected to induce similar sentiments as the religious songs (especially as the R_G_ condition). The selection was based on pre-experimental interviews with the cohort and visitations of services from the respective denominations. This song was the same for all participants.
**B**	Empty (*blank*)	This was a 4.5 min session where no music was played, hence an “empty” or “blank” condition. During this time, respondents were able to worship God and to try to induce the experience with no musical guidance or distraction. This session did not differ for any of the individuals.
**S_12_**	Twelve-tone song	This was a disharmonic twelve-tone song deliberately selected to throw the respondents off guard, which made it difficult for them to focus on God and to induce the experience. The song was the same for all participants.

**Table 2:** Analyses performed in the present research to find out how the physiological variables were affected by the religious experience during worship.

No.	Name	Analysis
**1**	Exploratory analysis	The data were inspected visually, and general associations were observed.
**2**	Testing the hypotheses (correlation analysis)	Parametric correlations were performed to find out if the peak experience (maximal values per experimental condition) were associated with the average HR and RR in the conditions.
**3**	Discerning the conditions (ANOVA and contrasts)	An analysis of variance (ANOVA) was applied to see if the physiology differed significantly during the experimental conditions, which was followed up by a contrast analysis to discover how exactly the conditions influenced RR and HR.
**4**	Covariance analysis (MANCOVA)	A multivariate analysis of covariance (MANCOVA) was performed to see if gender influences the relationship between the peak experience (maximal rating) and the physiology as a covariate.
**5**	Grouping analysis (factors and post hoc)	The variance of the data was reduced using a factor analysis. In a post hoc analysis, the emergent groups were screened to see if there were different religious experiences the groups made when engaging in worship with music.

Note: The values for the resting state C_RS_ were calculated by taking the mean from the two resting state conditions that have been placed at the beginning and the end of the experiments.

**Table 3:** Contrast coding for the present contrast analysis.

Condition		Contrasts (*Abbreviated with: C*)					
Name	Category	C1	C2	C3	C4	C5	C6
C_RS_	1	−6	0	0	0	0	0
S_12_	2	1	−5	0	0	0	0
B	3	1	1	−4	0	0	0
R_G_	4	1	1	1	1	0	−1
S_G_	5	1	1	1	−1	−1	0
R_S_	6	1	1	1	1	0	1
S_S_	7	1	1	1	−1	1	0

**Table 4:** Component matrix of the factor analysis with the relevant experiential, faith, and personality variables.

	Loadings			
Items	Factor 1 High Experiencers	Factor 2 Mid Experiencers	Factor 3 Low Experiencers	Communality
Maximal ratings (peak experience)	0.759	0.369	0.047	0.715
Experimental conditions	0.722	0.430	0.023	0.708
Evaluation (focus on God)	0.768	0.484	0.073	0.829
Mean RR	0.623	0.145	−0.095	0.418
Mean HR	0.338	−0.011	−0.076	0.120
Frequency of worshipping God with music	−0.356	0.683	0.209	0.638
Frequency of worshipping God without music	−0.171	0.291	0.086	0.122
Frequency of sensing the presence of God during musical worship	−0.274	0.457	0.086	0.291
General restlessness	0.230	−0.369	−0.214	0.235
Ability to stay calm	−0.279	0.430	0.062	0.267
Personality: Extraversion	0.142	−0.436	0.626	0.602
Personality: Agreeableness	−0.226	0.198	−0.023	0.091
Personality: Conscientiousness	−0.117	0.339	−0.667	0.574
Personality: Emotional Stability	−0.223	0.421	0.179	0.259
Personality: Openness to Experiences	0.222	−0.254	0.690	0.590

## Data Availability

The dataset generated during and/or analyzed during the current study is available from the corresponding author upon reasonable request.
